# Foot characteristics and mechanics in individuals with knee osteoarthritis: systematic review and meta-analysis

**DOI:** 10.1186/s13047-021-00462-y

**Published:** 2021-03-26

**Authors:** Rania N. Almeheyawi, Alessio Bricca, Jody L. Riskowski, Ruth Barn, Martijn Steultjens

**Affiliations:** 1 Institute of Applied Health Research, Glasgow Caledonian University, Glasgow, UK; 2 Department of Physical Therapy, Taif University, Taif, Saudi Arabia; 3 Research Unit for Musculoskeletal Function and Physiotherapy, Department of Sports Science and Clinical Biomechanics, University of Southern Denmark, Odense M, Denmark; 4 Department of Physiotherapy and Occupational Therapy, Næstved-Slagelse-Ringsted Hospitals, Region Zealand, Slagelse, Denmark; 5 Department of Podiatry and Radiography, Glasgow Caledonian University, Glasgow, UK

**Keywords:** Foot posture, Foot mechanics, Foot characteristics, Knee osteoarthritis

## Abstract

**Background:**

Foot characteristics and mechanics are hypothesized to affect aetiology of several lower extremity musculoskeletal conditions, including knee osteoarthritis (KOA). The purpose of this systematic review was to identify the foot characteristics and mechanics of individuals with KOA.

**Methods:**

Five databases were searched to identify relevant studies on foot characteristics and mechanics in people with KOA. Meta-analyses were performed where common measures were found across included studies. Included studies were evaluated for data reporting quality using the STROBE (STrengthening the Reporting of OBservational studies in Epidemiology) checklist.

**Results:**

Thirty-nine studies were included in this systematic review. Two studies reported participants with KOA had statistically significantly (*P* < 0.05) more pronated foot postures than those without. Meta-analyses for foot progression angle (FPA) and peak rearfoot eversion angle found no difference between those with and without KOA (FPA mean difference:-1.50 [95% confidence interval − 4.20-1.21]; peak rearfoot eversion mean difference: 0.71 [1.55–2.97]).

**Conclusion:**

A more pronated foot posture was noticed in those with KOA. However, it was not possible to establish a relationship between other foot characteristics or mechanics in people with KOA due to heterogeneity between the included study and limited number of studies with similar measurements. There is need for identifying common measurement techniques and reporting metrics when studying the foot in those with KOA.

## Background

Knee osteoarthritis (KOA) is a degenerative progressive joint disease characterized by chronic joint pain and stiffness, leading to the limitation of daily living activities and physical function [1–3]. KOA is estimated to affect 18% of adults over 45 years of age [4] and is a leading cause of functional disability [5]. Aetiology of KOA includes traumatic injury [6], genetics [7], obesity [8], and poor joint biomechanics, with poor biomechanics a likely cause of primary progressive KOA [9].

Given the important role of the foot in receiving and distributing forces during walking, foot characteristics and mechanics, including static foot posture and dynamic foot function, may significantly contribute to musculoskeletal conditions of the lower limb [10]. However, the specific associations between foot characteristics and mechanics and KOA [11] have not yet been investigated. Therefore, the primary purpose of this systematic review is to evaluate foot characteristics and mechanics in individuals with KOA and compare them to people without KOA. There were two aims of the study: 1) to provide an overview of the foot characteristics and mechanics that have been evaluated in the extant literature in people with KOA, and 2) to investigate whether foot characteristics and mechanics vary between people with and without KOA.

## Methods

This systematic review was submitted and approved through the PROSPERO registry of systematic reviews (CRD42015023946), and it followed the Preferred Reporting Items for Systematic Reviews and Meta-Analyses (PRISMA) guidelines [12].

### Search strategy and study selection

Five electronic databases were searched: MEDLINE, Web of Science, Current Nursing and Allied Health literature (CINAHL), Physical Education Index, and Physiotherapy Evidence Database (PEDro). The searches were conducted in May 2020, with no restrictions by language, year of publication or study design. The Medical Subject Headings (MeSH) search terms adopted were “foot” and “knee osteoarthritis” using the Boolean operator AND.

Studies were evaluated for relevance by applying specific inclusion and exclusion criteria (see Table 1). At the title stage, one reviewer (RA) eliminated publications, with a second reviewer (JLR) verifying the results. At the abstract stage, two reviewers (RA and JLR) independently reviewed abstracts for inclusion, and reference lists of prior KOA review articles were searched to include relevant studies. For manuscripts included following the abstract stage, full-text articles were obtained and independently reviewed for inclusion by reviewers (RA and JLR).

**Table 1:** Study inclusion criteria

Criteria	Description
Study design	Studies with cross-sectional data or intervention data if the baseline data were available.
Study participants	Studies were included if they recruited participants with KOA; where a control group was included, they had to be otherwise healthy and free from KOA.
Study outcome domains	Studies had to include objective measures of foot mechanics or foot characteristics to be eligible. Objective measures of foot mechanics or characteristics included, but were not limited to, foot progression angle, rearfoot eversion, Foot Posture Index and muscle activity. Further data could be obtained from participants in a barefoot or shod condition, provided the shod condition was without any foot orthoses.
Study results	Results had to provide quantitative data presented as mean and standard deviation or median and interquartile range clearly indicating if it was collected in a barefoot or shod condition.

### Data extraction

Data from the included manuscripts were extracted (RA) and checked (JLR). For each manuscript, the data extracted was as follows: the country, year of study, sample size, age, gender, body mass index (BMI), diagnostic and inclusion criteria for participants, footwear condition (i.e., barefoot, shod), foot-related outcome measures, and foot-related outcome data. For intervention studies, the baseline data were extracted for analysis. The level of agreement was determined using weighted kappa statistics for inclusion/exclusion.

### Assessment of study quality

Study quality of the information reported in the included manuscripts were based on the STROBE (STrengthening the Reporting of OBservational studies in Epidemiology) checklist criteria [13], which is a reliable quality rating tool for observational studies [14]. Each criterion was scored “Yes”, “No”, or not applicable (NA). A criterion received a “Yes” if it was applicable and met in the study, “No” if it was applicable but not met, and “NA” if it was not relevant to the study. The number of “Yes” criterion divided by the number of applicable criterions per manuscript yielded a percentage of the applicable STROBE criteria. Articles were dichotomized by their rating scores, with ≥65% regarded as high-quality studies, and < 65% deemed low-quality. The 65% cut-off point is similar to work conducted by Andrews et al. [15] in dichotomizing high and low quality studies. The 65% cut-off point is lower than the recommended cut-off point of 80% [16] as the reported foot characteristics and mechanics were often not the study’s primary outcome measure.

### Data analysis

Meta-analyses were performed to estimate the differences between the foot characteristics of participants, with and without KOA, for foot progression angle and peak rearfoot eversion angle. Mean differences (MD) with 95% confidence intervals (95% CI) were calculated. The standard deviation (SD) was extracted or estimated from the standard error of the mean, the 95% CI, *P* value, or other methods as recommended by the Cochrane Collaboration [17]. Meta-analyses were performed in STATA (16.1) using the ‘meta’ command. The effect sizes of the meta-analyses are reported in degrees.

## Results

**Fig. 1:**
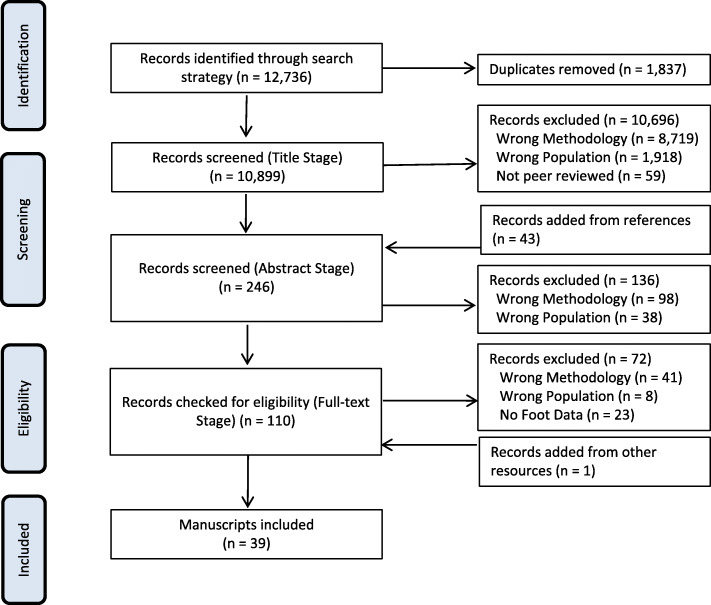
PRISMA flow chart diagram of the systematic review process

Following the implementation of the outlined search strategy, MeSH search yielded 12,736 articles, of which 1837 were duplicate publications (Fig. 1), leaving 10,899 articles for the title stage. Screening at the title stage excluded 10,696 of these articles, leaving 203 articles eligible for the abstract stage. At the abstract stage, 43 titles were added from reference lists and other sources, making a total of 246 articles eligible for the abstract stage, and 136 articles were excluded. A total of 110 articles were then reviewed at the full-text stage and 72 articles were excluded, while one article matching the eligibility criteria was added in the full-text stage from other sources, leaving 39 articles found to have evaluated foot characteristics and/or mechanics in individuals with KOA. Kappa agreement values between the reviewers were 0.79, 0.79, and 0.73 for the title, abstract, and full-text stage, respectively.

### Study characteristics

The included studies were published between 2006 and 2020 (Table 2). There were 25 observational studies [18–22, 25, 27, 29, 32, 33, 37–41, 43, 45–52, 56] and 14 intervention studies [23, 24, 26, 28, 30, 31, 34–36, 42, 44, 53–55]. The 39 studies included a total of 2260 participants. In the KOA groups, the sample sizes ranged from eight [37] to 123 [42] participants, with a mean study sample size of 57 participants. Twenty-two studies included a control population [18–22, 25, 27, 29, 31, 37–41, 45–47, 49–51, 54, 56], with sample sizes ranging from ten [37] to 80 [18] participants, and a mean control sample size of 17 participants. Thirty-two studies included both genders [18, 19, 21–24, 26–30, 33–35, 37–53, 55], while four studies were limited to women [20, 32, 54, 56]. Three studies failed to report gender characteristics [25, 31, 36].

**Table 2:** Study and participants’ characteristics (data reported as mean ± standard deviation)

No.	Author	Year published	Country	Subjects subgroups	No. of subjects (Men/ Women)	Age (years)	BMI (kg/m^**2**^)
1	Abourazzak et al. [18]	2014	Morocco	KOA	100 (21/79)	59.68 ± 7.64	30.89 ± 4.94
				Healthy control	80 (20/60)	48.66 ± 9.30	28.00 ± 3.81
2	Al-Zahrani and Bakheit [19]	2002	UK	KOA	58 (14/44)	71 ± 8.40	NR
				Healthy control	25 (10/15)	69 ± 7.29	NR
3	Anan et al. [20]	2015	Japan	KOA	20 (0/20)	69 ± 4.4	24.4 ± 2.8
				Healthy control	17 (0/17)	69.8 ± 4.3	21.3 ± 2.7
4	Arnold et al. [21]	2014	Australia	KOA	15 (7/8)	67.0 ± 8.9	30.7 ± 6.2
				Healthy control	15 (7/8)	68.2 ± 9.7	25.5 ± 5.3
5	Bechard et al. [22]	2012	Canada	KOA	20 (8/12)	55 ± 8	28.9 ± 3.0
				Healthy control	20 (12/8)	51 ± 8	25.9 ± 3.2
6	Booij et al. [23]	2020	Netherlands	Medial KOA only	30 (14/16)	62.7 ± 5.9	25.5 ± 2.7
7	Butler et al. [24]	2009	USA	KOA only	30 (13/17)	63.1 ± 6.8	33.8 ± 6.9
8	Butler et al. [25]	2011	USA	Medial KOA	15 (NR/NR)	66.2 ± 7.8	32.2 ± 7.9
				Lateral KOA	15 (NR/NR)	65.3 ± 6.4	30.4 ± 7.5
				Healthy control	15 (NR/NR)	56.3 ± 10.7	27.8 ± 5.7
9	Chapman et al. [26]	2015	UK	KOA only	70 (43/27)	60.3 ± 9.6	30.5 ± ± 4.9
10	Chang et al. [27]	2007	USA	KOA only	56 (23/33)	66.6 ± 8.6	29.0 ± 4.2
11	Charlton et al. [28]	2018	Canada	Medial KOA only	16 (6/10)	67.4 ± 9.3	24.6 ± 15.1
12	Elbaz et al. [29]	2017	Israel	KOA	63 (22/41)	64.2 ± 8.1	NR
				Healthy control	30 (21/9)	67.9 ± 8.9	NR
13	Erhart-Hledik et al. [30]	2017	Canada	Medial KOA only	10 (9/1)	65.3 ± 9.8	27.8 ± 3.0
14	Gardner et al. [31]	2015	USA	KOA	13 (NR/NR)	56.8 ± 5.2	26.6 ± 3.6
				Healthy control	11 (NR/NR)	50.0 ± 9.7	25.9 ± 5.4
15	Guler et al. [32]	2009	Turkey	KOA only	115 (0/115)	62.11 ± 8.72	32.91 ± 4.14
16	Guo et al. [33]	2007	USA	KOA only	10 (6/4)	64 ± 8	29.0 ± 5.6
17	Hinman et al. [34]	2012	Australia	KOA only	73 (28/45)	63.3 ± 8.4	27.7 ± 3.6
18	Hinman et al., [35]	2016	Australia	KOA only	81 (39/42)	63.3 ± 7.9	29.7 ± 3.7
19	Khan et al. [36]	2019	Malaysia	KOA only	20 (NR)	61.5 ± 8.63	NR
20	Krackow et al. [37]	2011	USA	KOA	8 (4/4)	59 ± 11.34	33.84 ± 6.90
				Healthy control	10 (5/5)	62.50 ± 4.17	28.44 ± 4.23
21	Levinger et al. [38]	2010	Australia	KOA	32 (16/16)	65.84 ± 7.57	29.97 ± 5.26
				Healthy control	28 (13/15)	65.22 ± 11.41	25.56 ± 3.95
22	Levinger et al. [39]	2012a	Australia	KOA	50 (27/23)	66.4 ± 7.6	29.6 ± 5.1
				Healthy control	28 (13/15)	65.1 ± 11.2	25.7 ± 3.9
23	Levinger et al. [40]	2012b	Australia	KOA	32 (16/16)	65.8 ± 7.5	29.9 ± 5.2
				Healthy control	28 (13/15)	65.2 ± 11.4	25.5 ± 3.9
24	Lidtke et al. [41]	2010	USA	KOA	25 (6/19)	60.2 ± 10.6	29.2 ± 4.6
				Healthy control	25 (12/13)	58.5 ± 9.1	26.6 ± 3.3
25	Nigg et al. [42]	2006	Canada	KOA only	123 (56/67)	57.4 ± 2.2	29.5 ± 1.6
26	Ohi et al. [43]	2017	Japan	KOA only	88 (30/58)	74.8 ± 7.58	24.3 ± 3.54
27	Paquette et al. [44]	2015	USA	KOA	13 (6/7)	62.5 ± 9	28.3 ± 6.5
				Healthy control	13 (5/8)	58.9 ± 8.3	23.9 ± 2.6
28	Park et al. [45]	2016	Canada	KOA	24 (7/17)	54 ± 7.3	26.1 ± 3.4
				Healthy control	24 (8/16)	52.4 ± 10.6	24.7 ± 3.2
29	Reilly et al. [46]	2006	UK	KOA	60 (25/35)	67.80 ± 8.09	NR
				Healthy control	60 (28/32)	64.92 ± 12.18	NR
30	Reilly et al. [47]	2009	UK	Medial KOA	20 (9/11)	63 ± 8.7	NR
				Healthy control	20 (4/16)	56 ± 7.3	NR
31	Rutherford et al. [48]	2008	Canada	KOA asymptomatic	50 (32/18)	53 ± 10	26 ± 4
				KOA mild to moderate	46 (20/26)	60 ± 9	31 ± 5
				KOA severe	44 (20/24)	67 ± 8	32 ± 5
32	Rutherford et al. [49]	2010	Canada	KOA	17 (10/7)	56 ± 8.8	29.8 ± 6.5
				Healthy control	20 (7/13)	46.5 ± 7.0	25.9 ± 4.8
33	Saito et al. [50]	2013	Japan	KOA	50 (10/40)	75	NR
				Elderly control	44 (8/36)	74	NR
34	Shakoor et al. [51]	2008	USA	KOA	27 (5/22)	54 ± 12	37.8 ± 8.6
				Healthy control	14 (5/9)	47 ± 14	29.8 ± 5.6
35	Simic et al. [52]	2013	Australia	KOA only	22 (9/13)	69.7 ± 9.0	28.4 ± 4.8
36	Tan et al. [53]	2020	Australia	KOA only	21 (7/14)	58 ± 8	27.0 ± 4.8
37	Trombini-Souza et al. [54]	2011	Brazil	KOA	21 (0/21)	6 5 ± 5	NR
				Healthy control	24 (0/24)	65 ± 4	NR
38	Van Tunen et al. [55]	2018	Australia	Medial KOA only	21 (9/12)	63.4 ± 7.0	29.8 ± 3.6
39	Zhang et al. [56]	2017	China	KOA	23 (0/23)	64.2 ± 6.6	23.3 ± 1.9
				Healthy control	23 (0/23)	62.1 ± 2.4	22.6 ± 1.8

*Abbreviations*: *KOA* knee osteoarthritis, *BMI* Body Mass Index, *NR* not reported

### Participant characteristics

#### Participant age

The mean age of the study participants was 61.5 years, ranging from 47 years [51] to 74 years [50] in the control groups, and 53 years [48] to 75 years [50] in KOA groups (Table 2).

#### Body mass index

In KOA groups, four studies reported a BMI mean of 18.5–24.9 kg/m^2^ (normal weight) [20, 28, 43, 56]; 19 studies reported participants’ mean BMI of 25–29.9 kg/m^2^ (overweight) [22, 23, 27, 30, 31, 33–35, 38–42, 44, 45, 49, 52, 53, 55]; eight studies reported the mean BMI of 30–34.9 kg/m^2^ (grade I obese) [18, 21, 24–26, 32, 37, 48]; and one study reported a mean BMI ≥35 kg/m^2^ [51] (grade II obese). Seven studies did not report the mean BMI of their participants [19, 29, 36, 46, 47, 50, 54]. In control groups, four studies reported a BMI mean of 18.5–24.9 kg/m^2^ (normal weight) [20, 44, 45, 56]; 12 studies reported participants’ mean BMI of 25–29.9 kg/m^2^ (overweight) [18, 21, 22, 25, 31, 37–41, 48, 51] and six studies did not report the mean BMI of their control participants [19, 29, 46, 47, 50, 54].

#### Participant eligibility criteria

The included studies evaluated foot characteristics and mechanics in those with KOA, yet four studies did not report the KOA diagnostic method used [19, 46, 47, 53]. Thirty-five studies diagnosed KOA severity using the Kellgren-Lawrence (KL) scoring system [18, 20–45, 48–52, 54–56].

### Assessment of study quality

Included studies were assessed for their reporting quality using the STROBE checklist criteria (Table 3). The percentages of STROBE criterion met ranged from 42% [19] to 84% [43]. Ten studies were categorized as high-quality studies [21, 25, 27, 35, 42–44, 47, 53, 55], while 29 studies scored less than 65% in relation to the applicable criteria on the STROBE checklist, and were therefore classified as low-quality studies [18–20, 22–24, 26, 28–34, 36–41, 45, 46, 48, 49, 51, 52, 54, 56].

**Table 3:** Assessment of study quality using the STROBE checklist

Item Number	Recommendations	Abourazzak et al., 2014 [18]	Al-zahrani amd Bakheit, 2002 [19]	Anan et al., 2015 [20]	Arnold et al., 2014 [21]	Bechard et al., 2012 [22]	Booij et al., 2020 [23]	Butler et al., 2009 [24]	Butler et al., 2011 [25]	Charlton et al. 2018 [28]	Chang et al., 2007 [27]	Chapman et al., 2015 [26]	Elbaz et al., 2017 [29]	Erhart-Hledik et al., 2017 [30]	Gardner et al., 2015 [31]	Guler et al., 2009 [32]	Guo et al., 2007 [33]	Hinman et al., 2012 [34]	Hinman et al., 2016 [35]	Khan et al., 2019 [36]	Krackow et al., 2011 [37]
1a	Abstract: study’s design in the title or the abstract	No	No	No	Yes	No	No	No	Yes	No	No	No	No	No	No	No	No	No	Yes	Yes	No
1b	Abstract: balanced summary	Yes	Yes	Yes	Yes	Yes	Yes	Yes	Yes	Yes	Yes	Yes	Yes	Yes	Yes	Yes	Yes	Yes	Yes	Yes	Yes
2	Introduction: background and rationale	Yes	Yes	Yes	Yes	Yes	Yes	Yes	Yes	Yes	Yes	Yes	Yes	Yes	Yes	Yes	Yes	Yes	Yes	Yes	Yes
3	Introduction: objectives, including hypotheses	Yes	Yes	Yes	Yes	Yes	Yes	Yes	Yes	Yes	Yes	Yes	Yes	Yes	Yes	Yes	Yes	Yes	Yes	Yes	Yes
4	Methods: study design early in the paper	Yes	No	No	Yes	No	Yes	No	Yes	No	No	No	Yes	Yes	No	No	No	No	Yes	Yes	No
5	Methods: setting, locations, and relevant dates, recruitment, data collection	No	Yes	Yes	Yes	Yes	Yes	Yes	Yes	Yes	Yes	Yes	Yes	Yes	Yes	Yes	Yes	Yes	Yes	Yes	Yes
6a	Methods: cohort eligibility criteria, follow-up	NA	NA	NA	NA	NA	NA	NA	NA	NA	Yes	NA	NA	NA	NA	NA	NA	NA	NA	NA	NA
6a	Methods: case-control: eligibility criteria of cases and controls	Yes	Yes	Yes	NA	NA	NA	NA	NA	NA	NA	NA	NA	NA	NA	NA	NA	NA	NA	NA	NA
6a	Methods: cross-sectional: eligibility criteria and methods of participants’ selection	NA	NA	Yes	Yes	Yes	Yes	Yes	Yes	Yes	NA	Yes	Yes	Yes	Yes	Yes	Yes	Yes	Yes	Yes	Yes
6b	Methods: cohort: number of exposed and unexposed	NA	NA	NA	NA	NA	NA	NA	NA	NA	NA	NA	NA	NA	NA	NA	NA	NA	Yes	NA	NA
6b	Methods: case-control: matching criteria	Yes	Yes	Yes	NA	Yes	NA	NA	NA	NA	NA	NA	Yes	NA	Yes	NA	NA	NA	NA	NA	NA
7	Methods: define outcomes, exposures, diagnostic criteria	Yes	Yes	Yes	Yes	Yes	Yes	Yes	Yes	Yes	Yes	Yes	Yes	Yes	Yes	Yes	Yes	Yes	Yes	Yes	Yes
8	Methods: sources of data, methods of assessment (measurement)	Yes	Yes	Yes	Yes	Yes	Yes	Yes	Yes	Yes	Yes	Yes	Yes	Yes	Yes	Yes	Yes	Yes	Yes	Yes	Yes
9	Methods: how bias addressed	No	No	No	No	No	No	No	No	No	Yes	Yes	No	No	No	No	No	No	Yes	Yes	No
10	Methods: power analysis	No	No	No	No	Yes	No	Yes	Yes	No	No	No	No	Yes	No	No	No	No	No	Yes	No
11	Methods: quantitative variables addressed	Yes	No	Yes	Yes	Yes	Yes	No	Yes	Yes	Yes	Yes	Yes	Yes	Yes	Yes	Yes	Yes	Yes	Yes	Yes
12a	Methods: statistical methods	Yes	Yes	Yes	Yes	Yes	Yes	Yes	Yes	Yes	Yes	Yes	Yes	Yes	Yes	Yes	Yes	Yes	Yes	Yes	Yes
12b	Methods: statistical subgroups and interactions	Yes	Yes	Yes	Yes	Yes	Yes	NA	Yes	Yes	Yes	Yes	Yes	Yes	Yes	No	Yes	NA	Yes	Yes	Yes
12c	Methods: how missing data addressed	NA	No	NA	No	No	No	NA	No	No	No	No	No	No	No	No	No	No	Yes	No	No
12d	Methods: cohort: how loss to follow-up addressed	NA	NA	NA	NA	NA	NA	NA	NA	NA	NA	NA	NA	NA	NA	NA	NA	NA	NA	NA	NA
12d	Methods: case-control: how matching of cases and controls addressed	No	No	No	NA	NA	NA	NA	NA	NA	NA	NA	NA	NA	NA	NA	NA	NA	Yes	NA	NA
12d	Methods: cross-sectional: sampling strategy	NA	NA	NA	Yes	No	No	Yes	Yes	No	NA	Yes	Yes	No	No	No	Yes	No	NA	No	No
12e	Methods: sensitivity analyses	No	No	No	Yes	No	Yes	No	Yes	No	No	No	No	No	No	No	No	No	Yes	No	No
13a	Results: numbers of individuals at each stage	Yes	No	Yes	Yes	Yes	No	Yes	Yes	No	Yes	Yes	Yes	Yes	Yes	Yes	Yes	Yes	Yes	No	Yes
13b	Results: reasons for non-participation at each stage	No	No	No	No	No	No	No	No	No	No	No	No	No	No	No	No	No	Yes	No	No
13c	Results: use of a flow diagram	No	No	No	No	No	No	No	No	No	No	No	No	No	No	No	No	No	Yes	No	No
14a	Results: characteristics of study participants	Yes	Yes	Yes	Yes	Yes	Yes	Yes	Yes	Yes	Yes	Yes	Yes	Yes	Yes	Yes	Yes	Yes	Yes	Yes	Yes
14b	Results: number with missing data	No	No	No	No	No	No	No	No	No	No	No	No	No	No	No	No	No	Yes	No	No
14c	Results: cohort: follow-up time	NA	NA	NA	NA	NA	NA	NA	NA	NA	Yes	NA	NA	NA	NA	NA	NA	NA	NA	NA	NA
15	Results: cohort: summary measures over time	NA	NA	NA	NA	NA	NA	NA	NA	NA	Yes	NA	NA	NA	NA	NA	NA	NA	NA	NA	NA
15	Results: case-control: summary measures of exposure	Yes	Yes	Yes	NA	NA	NA	NA	NA	NA	NA	NA	No	NA	NA	NA	NA	NA	Yes	NA	NA
15	Results: cross-sectional: numbers of events or measures	NA	NA	NA	Yes	Yes	Yes	Yes	Yes	Yes	NA	Yes	Yes	Yes	Yes	Yes	Yes	Yes	NA	Yes	Yes
16a	Results: unadjusted estimates	Yes	No	Yes	Yes	Yes	No	Yes	Yes	No	Yes	No	Yes	Yes	Yes	Yes	No	Yes	No	No	Yes
16b	Results: category boundaries	Yes	No	No	No	No	No	No	No	No	Yes	Yes	No	No	No	No	No	No	NA	No	No
16c	Results: translating relative risk into absolute risk	NA	No	No	Yes	No	NA	No	No	No	No	No	NA	No	NA	NA	No	No	NA	No	No
17	Results: other analyses (subgroups and interactions, and sensitivity)	Yes	No	No	No	No	No	No	No	No	Yes	No	Yes	No	Yes	No	No	Yes	Yes	No	Yes
18	Discussion: summarise key results with reference to study objectives	Yes	Yes	Yes	Yes	Yes	Yes	Yes	Yes	Yes	Yes	Yes	Yes	Yes	Yes	Yes	Yes	Yes	Yes	Yes	Yes
19	Discussion: limitations	No	No	Yes	Yes	No	Yes	No	Yes	Yes	No	Yes	Yes	No	Yes	No	No	Yes	Yes	Yes	Yes
20	Discussion: overall interpretation of results considering other relevant evidence	Yes	Yes	Yes	Yes	Yes	Yes	Yes	Yes	Yes	Yes	Yes	Yes	Yes	Yes	Yes	Yes	Yes	Yes	Yes	Yes
21	Discussion: generalisability of results	No	No	No	Yes	No	No	No	No	No	No	No	No	No	No	No	Yes	Yes	Yes	No	No
22	Funding: source of funding	No	No	No	No	Yes	Yes	No	Yes	Yes	Yes	Yes	No	Yes	Yes	No	No	Yes	Yes	Yes	No
%	Total percentage of successfully reported criteria in each study	61	42	58	72	58	58	53	72	50	66	63	64	59	63	48	53	61	94	61	56
Item Number	Recommendations	Levinger et al., 2010 [38]	Levinger et al., 2012a [39]	Levinger et al., 2012 [40]	Lidtke et al., 2010 [41]	Nigg et al., 2006 [42]	Ohi et al., 2017 [43]	Paquette et al., 2015 [44]	Park et al., 2016 [45]	Reilly et al., 2006 [46]	Reilly et al., 2009 [47]	Rutherford et al., 2008 [48]	Rutherford et al., 2010 [49]	Saito et al., 2013 [50]	Shakoor et al., 2008 [51]	Simic et al., 2013 [52]	Tan et al., 2020 [53]	Trombini-Souza et al., 2011 [54]	Van Tunen et al., 2018 [55]	Zhang et al., 2017 [56]	
1a	Abstract: study’s design in the title or the abstract	No	No	No	No	Yes	No	No	No	No	Yes	Yes	No	No	No	No	No	No	Yes	No	
1b	Abstract: balanced summary	Yes	Yes	Yes	Yes	Yes	Yes	Yes	Yes	Yes	Yes	Yes	Yes	Yes	Yes	Yes	Yes	Yes	Yes	Yes	
2	Introduction: background and rationale	Yes	Yes	Yes	Yes	Yes	Yes	Yes	Yes	Yes	Yes	Yes	Yes	Yes	Yes	Yes	Yes	Yes	Yes	Yes	
3	Introduction: objectives, including hypotheses	Yes	Yes	Yes	Yes	Yes	Yes	Yes	Yes	Yes	Yes	Yes	Yes	Yes	Yes	Yes	Yes	Yes	Yes	Yes	
4	Methods: study design early in the paper	No	No	No	No	Yes	Yes	No	No	Yes	Yes	Yes	No	No	Yes	No	Yes	No	Yes	No	
5	Methods: setting, locations, and relevant dates, recruitment, data collection	Yes	Yes	Yes	Yes	Yes	Yes	Yes	No	Yes	Yes	Yes	Yes	Yes	Yes	Yes	Yes	Yes	Yes	Yes	
6a	Methods: cohort eligibility criteria, follow-up	NA	NA	NA	NA	NA	NA	NA	NA	NA	NA	NA	NA	NA	NA	NA	NA	NA	NA	NA	
6a	Methods: case-control: eligibility criteria of cases and controls	NA	Yes	Yes	Yes	NA	NA	NA	NA	Yes	NA	NA	NA	NA	NA	NA	NA	NA	NA	Yes	
6a	Methods: cross-sectional: eligibility criteria and methods of participants’ selection	Yes	Yes	Yes	Yes	Yes	Yes	Yes	Yes	NA	Yes	Yes	Yes	Yes	Yes	Yes	Yes	Yes	Yes	NA	
6b	Methods: cohort: number of exposed and unexposed	NA	NA	NA	NA	NA	NA	NA	NA	NA	NA	NA	NA	NA	NA	NA	NA	NA	NA	NA	
6b	Methods: case-control: matching criteria	Yes	Yes	Yes	Yes	NA	NA	Yes	Yes	Yes	NA	NA	Yes	Yes	NA	NA	NA	NA	NA	Yes	
7	Methods: define outcomes, exposures, diagnostic criteria	Yes	Yes	Yes	Yes	Yes	Yes	Yes	Yes	Yes	Yes	Yes	Yes	Yes	Yes	Yes	Yes	No	Yes	Yes	
8	Methods: sources of data, methods of assessment (measurement)	Yes	Yes	Yes	Yes	Yes	Yes	Yes	Yes	Yes	Yes	Yes	Yes	Yes	Yes	Yes	Yes	Yes	Yes	Yes	
9	Methods: how bias addressed	No	No	No	No	No	Yes	No	No	No	Yes	No	No	No	No	No	No	No	No	No	
10	Methods: power analysis	No	No	No	No	Yes	No	Yes	No	Yes	Yes	No	No	No	No	Yes	Yes	Yes	No	No	
11	Methods: quantitative variables addressed	Yes	Yes	Yes	Yes	Yes	Yes	Yes	Yes	Yes	Yes	Yes	Yes	Yes	Yes	Yes	Yes	Yes	Yes	No	
12a	Methods: statistical methods	Yes	Yes	Yes	Yes	Yes	Yes	Yes	Yes	Yes	Yes	Yes	Yes	Yes	Yes	Yes	Yes	Yes	Yes	Yes	
12b	Methods: statistical subgroups and interactions	Yes	Yes	Yes	Yes	Yes	Yes	Yes	Yes	Yes	Yes	Yes	Yes	Yes	Yes	NA	Yes	Yes	Yes	Yes	
12c	Methods: how missing data addressed	No	No	No	No	Yes	No	No	No	No	No	No	No	No	No	No	No	No	No	No	
12d	Methods: cohort: how loss to follow-up addressed	NA	NA	NA	NA	Yes	NA	NA	NA	NA	NA	NA	NA	NA	NA	NA	NA	NA	NA	NA	
12d	Methods: case-control: how matching of cases and controls addressed	NA	No	No	No	NA	NA	No	No	No	NA	NA	No	No	NA	NA	NA	NA	NA	No	
12d	Methods: cross-sectional: sampling strategy	Yes	Yes	Yes	Yes	Yes	No	Yes	NA	NA	Yes	No	NA	NA	No	NA	Yes	No	No	NA	
12e	Methods: sensitivity analyses	No	No	No	No	No	Yes	Yes	No	Yes	Yes	No	No	No	No	No	Yes	No	Yes	No	
13a	Results: numbers of individuals at each stage	Yes	Yes	Yes	Yes	Yes	Yes	Yes	Yes	No	No	Yes	Yes	Yes	Yes	Yes	Yes	Yes	No	No	
13b	Results: reasons for non-participation at each stage	No	No	No	No	Yes	Yes	No	No	No	No	No	No	No	No	No	No	No	No	No	
13c	Results: use of a flow diagram	No	No	No	No	Yes	Yes	No	No	No	No	No	No	No	No	No	No	No	No	No	
14a	Results: characteristics of study participants	Yes	Yes	Yes	Yes	Yes	Yes	Yes	Yes	Yes	Yes	Yes	Yes	Yes	Yes	Yes	Yes	Yes	Yes	Yes	
14b	Results: number with missing data	No	No	No	No	Yes	Yes	No	No	No	No	No	No	No	No	No	No	No	No	No	
14c	Results: cohort: follow-up time	NA	NA	NA	NA	Yes	NA	NA	NA	NA	NA	NA	NA	NA	NA	NA	NA	NA	NA	NA	
15	Results: cohort: summary measures over time	NA	NA	NA	NA	Yes	NA	NA	NA	NA	NA	NA	NA	NA	NA	NA	NA	NA	NA	NA	
15	Results: case-control: summary measures of exposure	NA	NA	NA	NA	NA	NA	No	Yes	Yes	NA	NA	NA	NA	NA	NA	NA	NA	NA	Yes	
15	Results: cross-sectional: numbers of events or measures	Yes	Yes	Yes	Yes	Yes	Yes	Yes	Yes	NA	Yes	Yes	Yes	Yes	Yes	Yes	Yes	Yes	Yes	NA	
16a	Results: unadjusted estimates	Yes	Yes	Yes	Yes	No	Yes	Yes	Yes	No	Yes	Yes	Yes	No	Yes	Yes	Yes	No	Yes	No	
16b	Results: category boundaries	No	No	Yes	No	Yes	No	Yes	No	No	Yes	No	No	No	No	No	No	No	No	No	
16c	Results: translating relative risk into absolute risk	NA	No	No	NA	No	NA	No	NA	No	No	No	No	No	No	No	No	No	No	No	
17	Results: other analyses (subgroups and interactions, and sensitivity)	No	No	No	No	Yes	Yes	No	No	Yes	Yes	Yes	NA	No	Yes	No	No	No	Yes	No	
18	Discussion: summarise key results with reference to study objectives	Yes	Yes	Yes	Yes	Yes	Yes	Yes	Yes	No	Yes	Yes	Yes	Yes	Yes	Yes	Yes	Yes	Yes	Yes	
19	Discussion: limitations	No	Yes	Yes	Yes	No	Yes	Yes	Yes	No	Yes	Yes	Yes	Yes	No	Yes	Yes	No	Yes	No	
20	Discussion: overall interpretation of results considering other relevant evidence	Yes	Yes	Yes	Yes	No	Yes	Yes	Yes	Yes	Yes	Yes	Yes	Yes	Yes	Yes	Yes	Yes	Yes	Yes	
21	Discussion: generalisability of results	No	No	No	No	Yes	Yes	Yes	No	Yes	Yes	No	No	No	No	No	No	No	Yes	Yes	
22	Funding: source of funding	No	Yes	Yes	Yes	Yes	Yes	Yes	Yes	Yes	Yes	No	Yes	No	Yes	Yes	Yes	Yes	Yes	Yes	
%	Total percentage of successfully reported criteria in each study	56	60	63	62	83	84	69	58	61	81	63	63	52	63	60	69	50	69	48	

*Abbreviation*: *NA* not applicable

Among the common criterion not met included methods for addressing potential bias, with six meeting this criterion [26, 27, 35, 36, 43, 47]; study generalizability and external validity, with 11 meeting this criterion [27, 33–35, 42–44, 46, 47, 55, 56]; and sample size calculations provided, with 12 meeting this criterion [22, 24, 25, 30, 36, 42, 44, 46, 47, 52–54].

### Outcomes measures

Twenty-four studies included measures of participants taken while barefoot [18–21, 23, 27–29, 32, 36–41, 43, 44, 46–49, 51, 54, 55], while 14 were in shod conditions [22, 24–26, 30, 31, 33, 34, 42, 45, 50, 52, 53, 56] (Tables 4, 5 and 6). The majority of the studies (*n* = 24) used a three-dimensional (3D) motion analysis system and force platforms [19–28, 30, 31, 33, 34, 36, 37, 39, 40, 44, 48, 49, 52–54], whereas the rest (*n* = 14) used other measurement instruments including pressure plates [41], plantar pressure insoles [56], the Biodex system [42], static footprint [38], foot scanners [50], digital callipers [29], a dynamometer force system [45], a biothesiometer [51], and objective visual and manual measurements including foot posture index (FPI) [18, 47, 53, 55], goniometer [46], and lateral talometatarsal angle [32].

**Table 4:** Common foot variables in participants with KOA (data reported as mean ± standard deviation)

Foot variables	Study, year	Instrument- Shod condition	Results		*P*-value
			KOA	Controls	
Foot Progression Angle or toe-out degree (^0^)	Bechard et al., 2012	3D motion analysis system, force platform- Wearing lab shoes	6.2 ± 6.1	9.4 ± 5.0	0.68
	Booij et al., 2020	3D motion analysis system, force platform- Barefoot	−40.12 ± 4.80	No controls	NA
	Chang et al., 2007	3D motion analysis system, force platform- Barefoot	18.1 ± 8.4	No controls	NA
	Guo et al., 2007	3D motion analysis system, force platform- Wearing lab shoes	2.0 ± 6.8	No controls	NA
	Hinman et al., 2012	3D motion analysis system, force platform- Wearing lab shoes	−6.06 ± 5.56	No controls	NA
	Khan et al., 2019	3D motion analysis system, force platform- Barefoot	9.6 ± 3.7	No controls	NA
	Krackow et al., 2011	3D motion analysis system, force platform- Barefoot	8.58 ± 2.37	15.36 ± 2.12	NR
	Paquette et al., 2015	3D motion analysis system, force platform- Barefoot	13 ± 4	12.2 ± 3.5	0.82
	Rutherford et al., 2008	3D motion analysis system, force platform- Barefoot	7.5 ± 5	7.3 ± 5	NA
	Rutherford et al., 2010	3D motion analysis system, force platform- Barefoot	6.6 ± 7.3	4.9 ± 4.7	0.625
	Simic et al., 2013	3D motion analysis system- Wearing lab shoes	−4.5 ± 1.5	No controls	NA
	Trombini-Souza et al., 2011	3D motion analysis system, force platform- Barefoot	12.2 ± 6.74	13.1 ± 7.90	0.71
Peak rearfoot eversion (^0^)	Arnold et al., 2014	3D motion analysis system, force platform – Barefoot	5.3 ± 4.2	4.5 ± 5.0	0.850
	Butler et al., 2009	3D motion analysis system, force platform- Wearing lab shoes	3.5 ± 4.3	No controls	NA
	Butler et al., 2011	3D motion analysis system, force platform- Wearing lab shoes	6.2 ± 5.0	3.5 ± 2.7	0.01*
	Chapman et al., 2015	3D motion analysis system, force platform- Wearing lab shoes	3.51 ± 2.77	No controls	NA
	Erhart-Hledik et al., 2017	3D motion analysis system, force platform- Wearing lab shoes	13.9 ± 5.4	No controls	NA
	Levinger et al., 2012	3D motion analysis system, force platform- Barefoot	1.3 ± 5.2	2.3 ± 3.9	NR
	Nigg et al., 2006	Biodex system- Wearing lab shoes	41.9	No controls	NA
Peak rearfoot inversion (^0^)	Arnold et al., 2014	3D motion analysis system, force platform- Barefoot	1.4 ± 4.4	1.1 ± 4.2	0.708
	Levinger et al., 2012	3D motion analysis system, force platform- Barefoot	11.6 ± 5.2	14.9 ± 5.0	NR
	Nigg et al., 2006	Biodex system- Wearing lab shoes	45.1	No controls	NA
Pes planus prevalence (%)	Abourazzak et al., 2014	Visual observation (FPI)- Barefoot	42	22	0.03*
	Guler et al., 2009	Objective manual testing- Barefoot	38.3	No controls	NA
Foot pronation (difference in FPI)	Abourazzak et al., 2014	Visual observation (FPI)- Barefoot	1.5 ± 2.68	0.72 ± 2.63	0.05*
	Levinger et al., 2010	Visual observation (FPI)- Barefoot	2.46 ± 2.18	1.35 ± 1.43	0.022*

*Statistically significant *p*-value at 95% confidence interval

*Abbreviations*: *3D* three dimensional, *FPI* foot posture index, *KOA* knee osteoarthritis, *NA* not applicable, *NR* not reported

**Table 5:** Static foot variables in participants with KOA (data reported as mean ± standard deviation)

Study, year of publish	Foot variable (outcome)	Instrument- Shod condition	Results		P-value
			KOA	Controls	
Abourazzak et al., 2014 [18]	Prevalence of pes cavus (%)	Visual observation (FPI)- Barefoot	58	77	0.004*
Elbaz et al., 2017 [29]	Achilles tendon thickness (mm)	Digital caliper- Barefoot	17.1 ± 3.4	15.1 ± 3.1	0.009
Guler et al., 2009 [32]	Hallux valgus deformity (%)	Objective manual testing, radiography (x-ray)- Barefoot	22.60	No controls	NA
Hinman et al., 2016 [35]	FPI (n, %)	Visual observation (FPI)- Barefoot			
	Severely supinated		1 (1)	No controls	NA
	Supinated		0 (0)		
	Normal		44 (54)		
	Pronated		30 (37)		
	Severely pronated		6 (7)		
Levinger et al., 2010 [38]	Vertical navicular height	Objective manual testing, static footprint- Barefoot	0.23 ± 0.03	0.24 ± 0.03	0.542
	Navicular drop		0.02 ± 0.01	0.03 ± 0.01	0.019*
	Arch index		0.26 ± 0.04	0.22 ± 0.04	0.04*
Ohi et al., 2017 [43]	Hallux valgus angle (°)	3D footprint automatic (laser) measurement- Barefoot	13.6 ± 7.22	No controls	NA
	Presence of hallux valgus (%)		12.5		
	Navicular height (mm)		30.1 ± 6.75		
	Calcaneus angle relative to floor (°)		1.35 ± 5.09		
	Rear foot angle (°)		6.01 ± 3.76		
Reilly et al., 2006 [46]	Navicular height in sitting (cm)	Objective manual testing (goniometer)- Barefoot	5.22 ± 0.94	5.28 ± 0.89	0.005*
	Navicular height in standing (cm)		4.69 ± 0.83	4.73 ± 0.98	0.003*
Reilly et al., 2009 [47]	FPI**	Visual observation (FPI)- Barefoot	7.0 (−2 to 10)**	1.0 (−4 to 8)**	< 0.001*
	Ankle dorsiflexion during sitting (°)**	Objective manual testing using goniometer -Barefoot	9.0 (0 to 32)**	7.5 (0 to 15)**	< 0.001*
Shakoor et al., 2008 [51]	VPT (volts)	Biothesiometer, AP radiography- Barefoot			
	First MTPJ		15 ± 9.9	6.4 ± 3.3	< 0.001*
	Medial malleolus		22 ± 11.7	12.3 ± 5.2	0.001*
	Lateral malleolus		22.3 ± 10.5	10.4 ± 3.2	< 0.001*
Tan et al., 2020 [53]	FPI	Visual observation (FPI)- Midfoot and arch height mobility/arch indices- Barefoot	3 (1 to 7)	No controls	NA
	Arch height difference (mm)		8.8 ± 5.2		
	Midfoot width difference (mm)		8.9 ± 3.1		
	Foot mobility magnitude (mm)		14.8 ± 7.9		
Van Tunen et al., 2018 [55]	FPI (n, %)	Visual observation (FPI)- Barefoot Foot mobility magnitude calculation Navicular drop test			
	Normal (scores 0 to + 5)		9 (43)	No controls	NA
	Pronated (scores + 6 to + 9)		11 (52)		
	Highly pronated (scores greater + 9)		1 (5)		
	Foot mobility magnitude (mm)		9.6 ± 3.8		
	Navicular drop (mm)		7.6 ± 3.1		

*Statistically significant *p*-value at 95% confidence interval

** Data reported as median (interquartile range)

*Abbreviations*: *3D* three-dimensional, *FPI* foot posture index, *KOA* knee osteoarthritis, *MTPJ* metatarsophalangeal joint, *NA* not applicable, *NR* not reported, *SAI* Staheli arch index, *VPT* Vibratory perception threshold

**Table 6:** Dynamic foot variables in participants with KOA (data reported as mean ± standard deviation)

Study, year of publish	Foot variable (outcome)	Instrument- Shod condition	Results		*P*-value
			KOA	Controls	
Al-Zahrani and Bakheit 2002 [19]	Ankle plantar flexion in stance (°)**	3D motion analysis system, force platform- Barefoot	19.01 (15.90 to 22.70)**	30.88 (23.50 to 35.60)**	< 0.12
	Ankle plantar flexion in swing (°)**		27.76 (17.70 to 26.40)**	22.74 (15.90 to 22.70)**	< 0.02*
	Ankle moment (pre-swing) (Nm/kg)**		0.57 (0.36 to 0.78)**	0.79 (0.61 to 0.91)**	< 0.002*
	Ankle power (pre-swing) (Watt/k.)**		1.46 (0.53 to 2.31)**	3.86 (2.91 to 4.58)**	< 0.000*
Anan et al., 2015 [20]	Maximum ankle plantar flexion moment during STS (Nm/kg)	3D motion analysis system, force platform- Barefoot	0.36 ± 0.07	0.34 ± 0.07	0.343
	Mean ankle plantar flexion moment during STS (Nm/kg)		0.23 ± 0.06	0.24 ± 0.08	0.685
	Ankle planter flexion moment impulse during STS (Nms/kg)		0.47 ± 0.16	0.38 ± 0.15	0.072
Arnold et al., 2014 [21]	Hindfoot conronal plane ROM (^o^)	3D motion analysis system, force platform- Barefoot	10.9 ± 3.4	10.9 ± 4.3	0.562
Butler et al., 2009 [24]	Rearfoot eversion excursion (^o^)	3D motion analysis system, force platform- Wearing lab shoes	10.1 ± 2.8	No controls	NA
			−0.030 ± 0.034		
	Peak rearfoot eversion moment (Nm/kg*m)				
Butler et al., 2011 [25]	Peak rearfoot inversion moment (Nm/kg*m)	3D motion analysis system, force platform- Wearing lab shoes	−0.050 ±	− 0.062 ±	0.38
	Rearfoot eversion excursion (^o^)		0.045	0.03	0.96
			10.6 ± 5.6	10.2 ± 3.7	
Charlton et al. 2018 [28]	Foot rotation angle during natural walking:	3D motion analysis system- Barefoot			
	Ipsilateral foot (°)		−7.8 ± 7.9	No controls	NA
	Contralateral foot (°)		−8.4 ± 5.7		
Gardner et al., 2007 [31]	Planter flexion angle during cycling (^o^)	3D motion analysis system, force platform- Wearing lab shoes	−6.0 ± 8.5	− 8.9 ± 10.7	0.834
	Ankle eversion during cycling (^o^)		−6.8 ± 8.5	−13.2 ± 8.4	0.015*
	Internal rotation angle (^o^)		8.1 ± 7.1	9.2 ± 7.6	0.849
Guo et al., 2007 [33]	FPA during stair ascent (^o^)	3D motion analysis system, force platform- Wearing lab shoes	2.5 ± 6.6	No controls	NA
	FPA during stair descent (^o^)		11.3 ± 8.9		
Hinman et al., 2012 [34]	COP offset (mm)	3D motion analysis system, force platform- Wearing lab shoes	−5.6 ± 4.3	No controls	NA
Levinger et al.,2012a [39]	Ankle dorsiflexion (^o^)	3D motion analysis system, force platform-Barefoot	3.6 ± 3.3	2.4 ± 2.8	0.08
	Ankle adduction (^0^)		2.8 ± 1.9	4.2 ± 2.1	0.01*
	Toe clearance sensitivity in ankle (mm/degrees)		−0.1 ± 3.5	1.1 ± 4.5	0.05*
Levinger et al., 2012b [40]	Rearfoot frontal plane ROM (^o^)	3D motion analysis system, force platform- Barefoot	10.2 ± 3.3	12.5 ± 3.1	NR
	Rearfoot transverse plane ROM (^o^)		8.8 ± 4.7	10.0 ± 4.9	NR
	Internal rotation (^o^)		11.7 ± 6.3	15.4 ± 7.9	NR
	External rotation (^o^)		2.9 ± 5.8	5.4 ± 6.1	NR
Lidtke et al., 2010 [41]	COP index	Plantar pressure plate- Barefoot	−5.87 ± 5.6	−0.45 ± 3.45	< 0.001*
Nigg et al., 2006 [42]	Ankle plantar flexion (^o^)	Biodex system- Wearing lab shoes	50.6	No controls	NA
	Ankle dorsiflexion (^o^)		22.2		
Park et al., 2016 [45]	MVIC of ankle inversion muscle group (N/kg)	Force dynamometer- Wearing lab shoes	0.62 ± 0.26	0.86 ± 0.31	0.007*
Reilly et al., 2006 [46]	Ankle Plantar flexion in sitting (°)	Objective manual testing (goniometer)- Barefoot	50.72 ± 11.49	52.13 ± 10.94	0.788
	Ankle dorsiflexion in sitting (°)		10.07 ± 4.29	8.4 ± 3.71	0.000*
	Calcaneal angle in sitting (°)		2.02 ± 2.04	−0.25 ± 2.93	0.000*
Saito et al., 2013 [50]	Partial foot pressure per body weight (%)	Plantar pressure sensor insoles during walking- Wearing lab shoes			
	Heel		27.1 ± 11.2	41.7 ± 8.5	< 0.001*
	Central		33.1 ± 11.2	16.5 ± 13.8	< 0.001*
	Metatarsal		12.4 ± 7.9	12.1 ± 6.7	> 0.001
	Hallux		1.5 ± 2.2	3.5 ± 3.0	< 0.001*
	Lateral toes		1.2 ± 1.7	2.5 ± 2.1	> 0.001
Tan et al., 2020 [53]	Peak dorsiflexion angle in stance (°) during walking	3D motion analysis system, force platform-Wearing lab shoes	14.9 ± 3.2	No controls	NA
	Peak dorsiflexion moment (Nm/kg) during walking		0.15 ± 0.27		
	Peak dorsiflexion angle in stance (°) stair ascent / descent.		9.7 ± 4.4		
	Peak dorsiflexion moment (Nm/kg) stair ascent / descent.		1.08 ± 0.22		
	Weight bearing ankle joint dorsiflexion ROM (cm)	Knee to wall test	9.1 ± 3.2		
Zhang et al., 2017 [56]	Contact area (cm^2^)	Plantar pressure sensor insoles during walking- Wearing lab shoes			
	Heel		28.9 ± 2.9	28.6 ± 1.7	0.982
	Midfoot		41.5 ± 5.8	36.5 ± 7.3	0.043*
	1st MTPJ		13.8 ± 1.6	13.1 ± 1.3	0.875
	2nd MTPJ		13.6 ± 0.8	13.2 ± 1.3	0.922
	3rd-5th MTPJ		12.7 ± 0.6	12.8 ± 0.3	0.986
	Hallux		7.1 ± 1.7	6.6 ± 1.6	0.684
	Lesser toes		10.3 ± 1.1	10.8 ± 0.4	0.988
	Maximum force (%BW)				
	Heel		69.5 ± 15.2	67.1 ± 11.3	0.817
	Midfoot		30.3 ± 7.1	23.6 ± 7.4	0.43
	1st MTPJ		32.3 ± 7.1	26.5 ± 6.2	0.037*
	2nd MTPJ		35.2 ± 9.1	30.3 ± 5.1	0.041*
	3rd-5th MTPJ		17.7 ± 5.4	16.7 ± 4.9	0.843
	Hallux		14.3 ± 6.5	13.5 ± 5.6	0.901
	Lesser toes		12.0 ± 4.7	12.6 ± 3.2	0.973
	Plantar pressure (kPa)				
	Heel		252.9 ± 52.5	243.7 ± 52.5	0.581
	Midfoot		132.8 ± 28.3	116.5 ± 30.0	0.031*
	1st MTPJ		295.1 ± 100.4	224.3 ± 62.4	0.024*
	2nd MTPJ		273.8 ± 103.9	244.6 ± 56.1	0.183
	3rd-5th MTPJ		156.1 ± 43.1	157.9 ± 49.3	0.981
	Hallux		231.9 ± 77.6	219.6 ± 79.4	0.531
	Lesser toes		139.4 ± 49.4	142.9 ± 44.9	0.801

*Statistically significant *p*-value at 95% confidence interval

** Data reported as median (interquartile range)

*Abbreviations*: *3D* three-dimensional, *%BW* percent bodyweight, *AP* anteroposterior, *COP* centre of pressure, *KOA* knee osteoarthritis, *NA* not applicable, *NR* not reported, *MVIC* maximum voluntary isometric contraction, *MTPJ* metatarsophalangeal joint, *ROM* range of motion, *STS* sit-to-stand

A wide range of foot characteristics and mechanics were reported in the included studies. The most common foot-related outcomes investigated and reported were foot progression angle (FPA) or toe-out degree (*n* = 12) [22, 23, 27, 33, 34, 36, 37, 44, 48, 49, 52, 54], and peak rearfoot eversion angle (*n* = 7) [21, 24–26, 30, 40, 42]. Other outcome measures included the prevalence of pes planus among participants with KOA measured with reference to the medial arch index and the lateral talometatarsal angle [18, 32], and foot pronation measured by foot posture index (FPI) [18, 38]. One study measured partial foot pressure percentage by body weight [50], and another measured plantar load during walking [56].

#### Foot progression angle (toe-out degree)

**Fig. 2:**
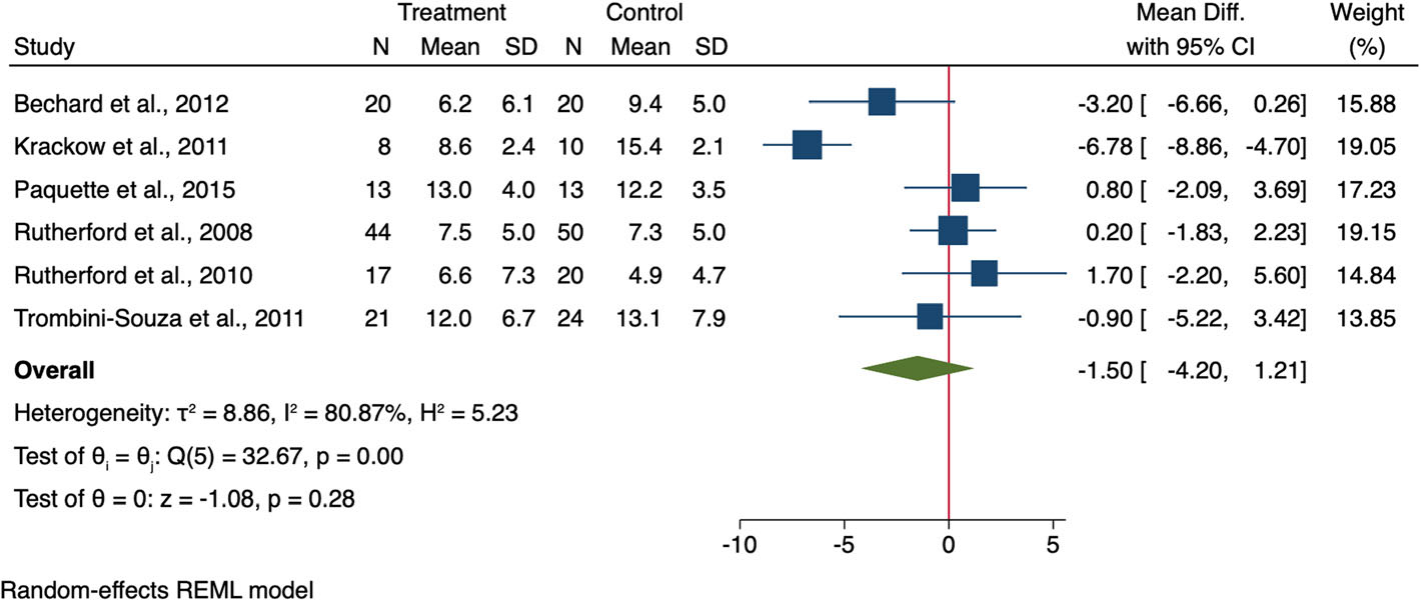
Forest plot for the differenuihjhjce in FPA during walking between KOA people and healthy controls. 95% CI = 95% Confidence Interval, SD = standard deviation

Twelve studies measured and reported FPA [22, 23, 27, 33, 34, 36, 37, 44, 48, 49, 52, 54]. Six studies recruited both KOA and control groups and compared the findings between them [22, 37, 44, 48, 49, 54]. The FPA meta-analysis showed no difference between participants with and without KOA (MD: -1.50, 95% CI − 4.20 to 1.21) (Fig. 2). Six other studies recruited KOA participants without a control group [23, 27, 33, 34, 36, 52], and three of these reported negative values for FPA [23, 34, 52], meaning that KOA participants walked with in-toeing gait, while the other three studies reported positive values of FPA [27, 33, 36] [27, 33, 36], meaning that KOA participants tended to walked with a toe-out gait.

#### Peak rearfoot eversion angle

**Fig. 3:**
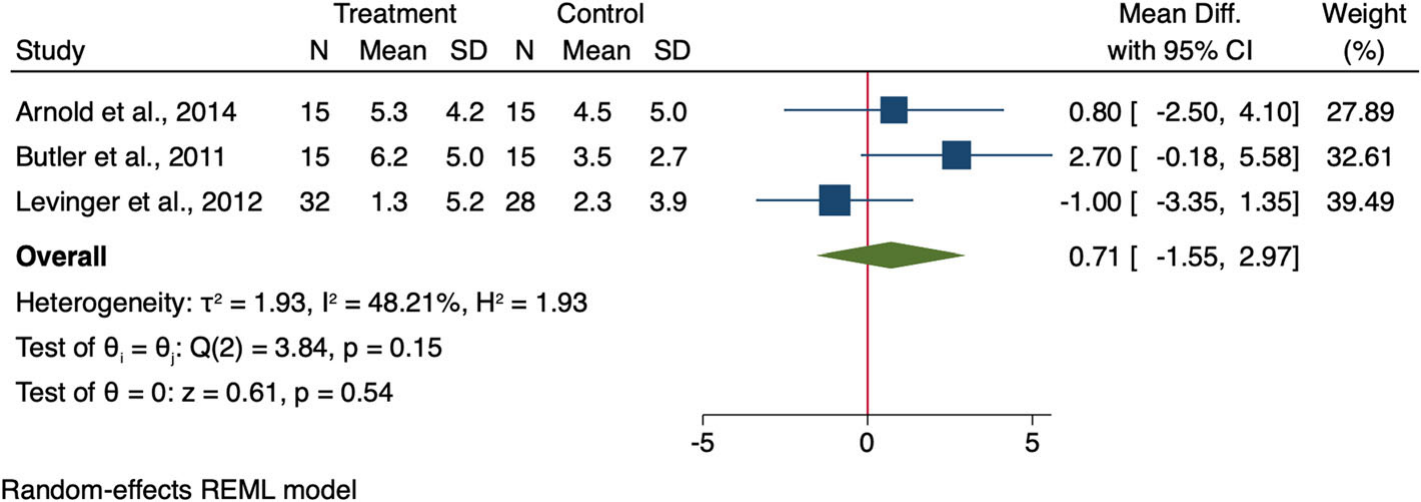
Forest plot for the difference in peak rearfoot eversion angle during walking between KOA people and healthy controls. 95% CI = 95% Confidence Interval, SD = standard deviation

Seven studies measured peak rearfoot eversion angle in individuals with KOA [21, 24–26, 30, 40, 42] using 3D motion analysis systems (in weight bearing position during walking) [21, 24–26, 30, 40], and Biodex (non-weight bearing, in sitting position) [42]. Four studies recruited a KOA group only [24, 26, 30, 42], while three studies compared data to those without KOA [21, 25, 40] (Table 4). A meta-analysis of these studies showed no significant difference in peak rearfoot eversion angle during walking between groups (MD: 0.71, 95%CI − 1.55 to 2.97) (Fig. 3).

#### Foot posture

FPI was reported in six studies [18, 35, 38, 47, 53, 55]. However, the study outcomes were not presented comparably between these studies, limiting the possibilities of meta-analysis. Two studies measured differences in foot posture using FPI in KOA and non-KOA populations [18, 38]. Both of them noted that participants with KOA had statistically significant (*P* < 0.05) highly pronated foot postures, with a difference of 0.78 [18] and 0.61 [38] between the groups (Table 4). Four additional studies measured FPI in individuals with KOA [35, 47, 53, 55], with the results reported here in Table 5 as they were measured differently, with two reporting results as median and interquartile ranges [47, 53] and two categorising and reporting the prevalence of individuals into categories. The first study categorised individuals into three categories: normal, pronated, or highly (severely) pronated [55], while the other study added two categories: supinated, and severely supinated [35]. The highest prevalence in both studies was in the pronated foot posture category, with 52% of participants (*N* = 11) in one study [55] and 37% (*N* = 30) in the other [35] (Table 5).

#### Pes planus

Two studies reported on the prevalence of pes planus in individuals with KOA. Pes planus was measured with reference to the medial arch index in one study, and it showed a statistically significant greater prevalence of pes planus in participants with KOA (42% vs. 22%) [18]. Another study measured pes planus by the lateral talometatarsal angle, where it was defined as an angle > 4°, and reported that 38.3% of participants with KOA had pes planus [32].

#### Other outcomes

Other foot characteristics and mechanics measured in individuals with KOA were divided into two categories and reported in two different tables: static foot variables (Table 5) and dynamic foot variables (Table 6). The medial arch of the foot was assessed and reported in four studies using different methods (vertical navicular height, navicular drop, and arch index), with different tools (arch index, static footprint, goniometer, and navicular drop test). Of those four studies, two studies compared the results of the KOA group to a control group [38, 46]. When participants with KOA were compared to those without, they were found to have a more significant navicular drop (0.03 ± 0.01 vs 0.02 ± 0.01), a significantly greater arch index (0.26 ± 0.04 vs 0.22 ± 0.04) [38], and significantly lower navicular height in sitting (5.22 ± 0.94 cm vs 5.28 ± 0.89 cm) [46] and standing (4.69 ± 0.83 cm vs. 4.73 ± 0.98 cm) [46].

Plantar pressure was measured during walking while wearing plantar pressure sensor insoles embedded inside lab shoes in two studies [50, 56]. One study [50] assessed and reported the percentage of partial foot pressure per body part, and reported that plantar pressure was statistically lower in participants with KOA compared to those without KOA in the heel (27.1 ± 11.2% vs. 41.7 ± 8.5%), and hallux (1.5 ± 2.2% vs. 3.5 ± 3.0%), and statistically greater at the midfoot (central) (33.1 ± 11.2% vs. 16.5 ± 13.8%) [50]. In the other study [56], a significantly greater plantar pressure was reported in the midfoot (132.8 ± 28.3 kPa vs. 116.5 ± 30.0 kPa), and the first metatarsophalangeal joint (295.1 ± 100.4 kPa vs. 224.3 ± 62.4 kPa) when compared to a control population [56].

One study [51] investigated the vibratory perception threshold (VPT) in specific foot areas and reported significant deficits in vibratory sensation in participants with KOA. Compared to participants without KOA, those with KOA demonstrated significantly greater VPT in the first metatarsophalangeal joint (15 ± 9.9 V vs. 6.4 ± 3.3 V), medial malleolus (22 ± 11.7 V vs. 12.3 ± 5.2 V), and lateral malleolus (22.3 ± 10.5 V vs. 10.4 ± 3.2 V) [51]. Another study which explored Achilles tendon thickness reported significantly thicker tendons in the KOA group compared to the control [29] (17.1 mm vs. 15.1 mm), with thickness associated positively with KOA severity.

## Discussion

The purpose of this review was to evaluate foot characteristics and mechanics in individuals with KOA and compare them to people without KOA where possible. Variations in foot characteristics and mechanics in people with KOA were found in the included studies. These variations included differences in FPA, peak rearfoot eversion angle, pronated foot posture, and incidence of pes planus in people with KOA. Several studies compared foot characteristics and mechanics in individuals with KOA to those without KOA; however measurement techniques and outcome measures were not homogenous across studies. Therefore, meta-analyses were conducted on two foot variables only, FPA and peak rearfoot eversion angle. However, these revealed no statistical difference in FPA or peak rearfoot eversion angle. The results across the included studies were inconsistent, a situation which can be attributed to three main reasons: 1) several studies had no control group without KOA, limiting the ability to report between group differences; 2) studies employed different measurement techniques or methods of reporting, limiting the ability to combine data in meta-analyses; and 3) foot characteristics or mechanics were reported by only one study (e.g., VPT, prevalence of hallux valgus deformity, Achilles tendon thickness), making it impossible to draw robust conclusions. Therefore, further work is needed to fully understand the differences in foot characteristics and mechanics in individuals with KOA.

Results of the present work suggest that the prevalence of pes planus and pronated foot posture is higher among participants with KOA. Zhang et al. (2017) reported significantly greater plantar pressure in the midfoot in those with KOA compared to those without. The increase of midfoot and central plantar pressure aligns with the increased incidence of pes planus [18, 32] and greater foot pronation [18, 38] associated with KOA. Further, the positive association noted between pes planus and lower vertical navicular height [38] may explain the high pressure in the midfoot area and the absence of a medial longitudinal arch in the foot [50]. The greater peak rearfoot eversion angles evident in individuals with KOA [21, 25, 31] also align with the reported FPA differences between those with and without KOA [22, 34, 37, 52, 54], as these measurements are hypothesised to influence each other biomechanically.

As the included studies measured foot characteristics and mechanics in those with KOA at a single time point, it is unclear if foot posture or incidence of pes planus is a cause or effect of KOA. Nonetheless, the presence of the biomechanical foot differences (pronated foot posture, greater peak rearfoot eversion angle, and incidence of pes planus) associated with KOA highlight the importance of the kinetic chain and biomechanical influence of one joint on another, which may indicate that foot characteristics may be related to KOA progression. However, further longitudinal studies are required to confirm this. As foot posture and foot function have previously been associated with knee joint loading [38, 57], a cause of primary progressive KOA [9], it is possible that changing the foot posture or function may be an appropriate intervention for KOA.

Conservative interventions targeting a biomechanical change to address KOA have included foot-related interventions [58, 59]. The most common foot-related interventions used to manage KOA are gait modifications and lateral wedge insoles [58]. Toe-out gait has been widely deployed as a conservative intervention in order to reduce knee adduction moment (KAM) and symptoms in people with KOA [59]. Walking with a greater toe-out angle as a mechanical intervention changes the knee joint load in individuals with KOA, shifting the KAM into a flexion moment and reducing knee pain [60]. Furthermore, a greater toe-out degree during walking has been associated with a reduced likelihood of disease progression in participants with KOA for over 18 months [27]. Therefore, this intervention can be limited to targeting people with KOA who walk with a toe-in gait pattern. However, the findings of this systematic review also revealed a diversity in walking patterns among people with KOA (toe-in vs. toe-out gait); thus, this intervention cannot be applied widely in people with KOA.

Lateral wedge orthoses are another common foot-related intervention for KOA [58]. A recent systematic review and meta-analysis demonstrated a reduction in knee joint load, reported as a significant small reduction in first peak of external KAM (standardized mean difference [SMD]: − 0.19; 95% confidence interval [95% CI] -0.23, − 0.15) and second peak external KAM (SMD -0.25; 95% CI -0.32, − 0.19) with a low level of heterogeneity (I^2^ = 5 and 30%, respectively) and small but favourable reduction in knee adduction angular impulse during walking in people with KOA (SMD = − 0.14; 95% CI -0.21, − 0.07, I^2^ = 31%) [58]. However, the biomechanical changes reported as resulting from lateral wedge orthoses were considered minimal, thus limiting the efficacy of this intervention [58]. Furthermore, the impact of this intervention is still unknown for people with KOA who have pronated foot posture as lateral wedge orthoses were reported to significantly increase subtalar joint valgus moment [61]. Therefore, defining foot characteristics and mechanics in individuals with KOA is extremely important, as doing so can play an essential role in selecting the most appropriate foot-related interventions to fit the individual’s own foot characteristics and mechanics.

This systematic review has identified several gaps and areas where future research is needed. Intrinsic foot muscle strength, which affects gait and balance [62], remains an unknown characteristic in the KOA population. Future work evaluating the association between foot muscle strength and KOA may prove beneficial in determining if foot strength or its improvement may be an effective KOA intervention. Further, only one study [51] to date has investigated and reported a loss of vibratory sensation in the foot and ankle with KOA, a measure also affecting gait [63]. Understanding if there is a loss in vibratory sense loss or proprioception as well as how it affects those with KOA may also inform the type of rehabilitation deemed appropriate for this population. It has been suggested that poor neuromuscular control affects injury risk and prevention [64], and neuromuscular control has been associated with KOA severity [65]. Therefore, improving foot neuromuscular control may potentially lessen the risk of knee injury and decrease the impact of KOA.

### Strengths and limitations

As with any study, the systematic review and meta-analyses presented here should be evaluated with respect to their strengths and limitations. This review set out a wide range of foot characteristics and mechanics in people with KOA. However, most of the measures were only reported in one or two studies with a small sample of participants, which may limit their generalisability to the wider KOA population. Further, this study has evaluated foot characteristics and mechanics in individuals with KOA and suggested a potential relationship between some of the foot measures and KOA. However, the potential cause and effect relationship of foot characteristics and mechanics outcome measures to KOA is still unknown, as this work has reported foot- related data collected at one time point from observational studies, or data at baseline from intervention studies. Future researchers are advised to investigate the relationship between KOA and foot characteristics and mechanics in more depth via longitudinal studies.

One strength of this study is its robust design, which allowed for the breadth of foot characteristics published to be included in the systematic review and meta-analysis, providing a strong background for researchers to develop longitudinal and intervention studies. However, the wide variety of techniques used to measure similar outcomes prevented the possibility of conducting multiple meta-analyses. Therefore, future studies are advised to develop and follow standardized techniques with which to measure foot characteristics and mechanics in order to facilitate further meta-analyses.

The foot characteristics and mechanics reported in this systematic review were assessed and measured using a range of specific measurements. These could be divided into two categories: 1) laboratory-based measurement (e.g., 3D motion capture, static footprint, force platform, and Biodex); and 2) visual observation and objective manual measurements (e.g., navicular drop test, knee to wall test, FPI, Staheli arch index, and digital caliper). Many of the included studies omitted to provide sufficient details on how the measurements were taken. Moreover, due to the heterogeneity in measurement methods used to investigate foot characteristics and mechanics between the included studies, the process of pooling results for comparison was limited.

One of the limitations identified during this review was the lack of quality in the included studies, as only ten studies attained 65% on the STROBE checklist and could thus be considered high-quality studies. A lower cut-off point of 65% was utilized during the assessment of study quality because foot characteristics and mechanics were not generally the primary outcome measure in the included studies; thus, a cut-off point higher than 65% would not have been achievable by the included studies.

## Conclusion

In conclusion, despite the large body of prior research investigating foot characteristics and mechanics in individuals with KOA, many studies lacked a comparison group without KOA. Five foot characteristics and mechanics measures were commonly reported in the included studies (FPA, rearfoot peak eversion angle, peak rearfoot inversion angle, foot posture, and prevalence of pes planus). A more pronated foot posture was noticed in the presence of KOA. Further, of these five common foot characteristics and mechanics, two were of similar design, enabling a meta-analysis to be conducted - FPA and peak rearfoot eversion angle. Meta-analysis of these two variables demonstrated no significant differences between participants with and without KOA. Thus, the implications of the present work suggest a need to adopt and adhere to unified measurement techniques of common foot characteristics and mechanics to make meta-analyses more viable. Lastly, longitudinal studies are needed to identify the potential causal relationship between foot characteristics and mechanics and KOA in people with KOA.

## Data Availability

Dataset generated and analysed during the current study are available from the corresponding author on request.

## Abbreviations

3D: Three-dimensional
BMI: Body mass index
FPA: Foot progression angle
FPI: Foot posture index
KAM: Knee adduction moment
KL: Kellgren-Lawrence scoring system
KOA: Knee osteoarthritis
MD: Mean difference
mm: Millimetre
SD: Standard deviation
SMD: Standardized mean difference
STROBE: Strengthening the reporting of observational studies in epidemiology
ROM: Range of motion
VPT: Vibratory perception threshold
